# Diffusion-Tensor Magnetic Resonance Imaging for Hand and Foot Fibers Location at the Corona Radiata: Comparison with Two Lesion Studies

**DOI:** 10.3389/fnhum.2014.00752

**Published:** 2014-09-29

**Authors:** Dong-Hoon Lee, Cheolpyo Hong, Bong-Soo Han

**Affiliations:** ^1^Division of Magnetic Resonance Research, Department of Radiology, Johns Hopkins University School of Medicine, Baltimore, MD, USA; ^2^Center for Medical Metrology, Korea Research Institute of Standards and Science (KRISS), Daejeon, South Korea; ^3^Department of Radiological Science, College of Health Science, Yonsei University, Wonju, South Korea

**Keywords:** corticospinal tract, corona radiata, diffusion-tensor imaging, functional imaging, motor pathway, somatotopy

## Abstract

The corticospinal tract is the motor pathway in the human brain, and corona radiata (CR) is an important location to diagnose stroke. We detected hand and foot motor fiber tracts in the CR to investigate accurate locations using diffusion-tensor imaging (DTI) and functional imaging. Ten right-handed normal volunteers participated in this study. We used a probabilistic tracking algorithm, a brain normalization method, and functional imaging results to set out region of interests. Moreover, our results were compared to previous results of lesion studies to confirm their accuracy and usefulness. The location measurements were performed in two index types; anteriority index on the basis of the anterior and posterior location of lateral ventricle and laterality index on the basis of the left and right location. The anteriority indices were 56.40/43.2 (hand/foot) at the upper CR and lower CR 40.72/30.90 at the lower CR. The measurements of anteriority and laterality of motor fibers were represented as anteriority index 0.40/0.31 and laterality index 0.60/0.47 (hand/foot). Our results showed that the hand and foot fibers were in good agreements with previous lesion studies. This study and approaches can be used as a standard for DTI combined with lesion location studies in patients who need rehabilitation or follow-up.

## Introduction

The corticospinal tract (CST) is a motor pathway in the human brain. It originates from the cerebral cortex and travels via the corona radiata (CR), posterior limb of the internal capsule (PLIC), and pons (Holodny et al., 2005a,b; Jang, 2011; Masri, 2011). Detailed information on somatotopy of the CST would enable us to predict the motor outcome, and set up the scientific management strategy of the patients with brain injury (Jang, 2011; Kwon et al., 2011b). Particularly, the somatotopic location of CST in the CR is an important consideration for the patients with brain injury because the lesions of brain injury are commonly located on the CR. Recently, many studies have been done for elucidating the somatotopic location of CST using diffusion-tensor tractography (DTT), which derived from diffusion-tensor image (DTI), combined with other technical methods such as functional magnetic resonance imaging (fMRI) (Holodny et al., 2005a,b; Hagmann et al., 2006; Smits et al., 2007; Mukherjee et al., 2008; Mancini et al., 2009; Suzuki et al., 2009; Masri, 2011; Jang, 2011; Lee et al., 2012; Seo et al., 2012).

In this study, we attempt to investigate the accurate locations for CSTs of both the hand and foot in the CR. Although there are some DTT studies that have been performed on the CST anatomical location as mentioned above, our study has novelty points compared to the other studies for accurate CST location measurements as follows. First, in the two CR level, we used a probabilistic fiber tracking algorithm to minimize the effect of the interruption of crossing fibers for the CST, and fMRI activation areas on the hand and foot motor cortices to first identify the upper seed regions within the primary motor cortex (Guye et al., 2003; Hong and Jang, 2011). Second, we used fiber normalization method using brain template to avoid individual variation. Third, the location of the CST’s measurements that contain information related to two previous lesion studies were used in the measurement as a reference (Kim and Pope, 2005; Song, 2007). Our results were also compared with the results of these previous lesion studies.

## Materials and Methods

### Subjects

Ten right-handed normal volunteers (seven males, three females; mean age 42.5 years) with no history of a neurological disorder participated in this study. All subjects provided signed informed consent prior to the commencements of the study. The study protocol was approved by our local institutional review board.

### Data acquisition

Diffusion-tensor imaging and fMRI datasets were obtained using a 1.5-T MR scanner (Gyroscan Intera, Philips Healthcare, Best, The Netherlands) with a six-channel phased array sensitivity encoding (SENSE) head coil based on a single-shot echo planar imaging (EPI) pulse sequence. The fMRI data had imaging parameters as follows: field of view (FOV) = 210 mm, time of repetition (TR)/time of echo (TE) = 2000/60 ms, matrix = 64 × 64, slice thickness = 5 mm, and SENSE factor = 2. The motor task was performed using grasp-release finger and toe movements at a frequency of 1 Hz according to the block paradigm (rest/activation = 21/21 s, repeated three times). The DTI data had imaging parameters as follows: FOV = 221 mm, TR/TE = 10,726/75 ms, matrix = 128 × 128, and slice thickness = 2.3 mm. The diffusion weighting gradient was applied along 32 distinct directions with a *b*-value of 1000 s/mm^2^.

### Data analysis

The fMRI data were analyzed using SPM2 (The Wellcome Department of Cognitive Neurology, London, UK) with family-wise error (FWE) set at *p* < 0.05. In the process of fMRI data analysis, all images were registered based on non-diffusion-weighted images (*b* = 0 s/mm^2^) in order to overlay their motor-related activation results and use the region of interest (ROI) setting for fiber tracking. An 8 mm full-width at half-maximum (FWHM) Gaussian smoothing kernel was used to increase signal-to-noise (SNR) and validity of statistics (Guo and Pagnoni, 2008;Koch et al., 2012;Korgaonkar et al., 2013).

**Figure 1:**
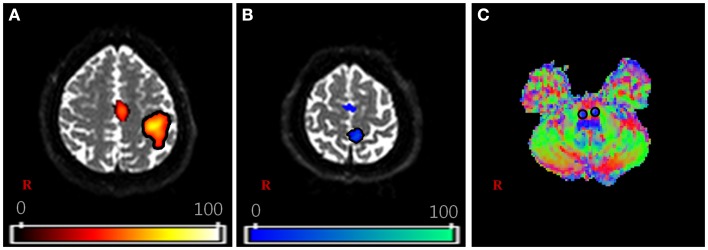
**The region of interest (ROI) setting for fiber tracking**. **(A,B)** are shown the activation area of functional MRI with finger and toe movement, respectively. **(C)** is shown the part of lower portion of the pons on the V1 image of diffusion-tensor image (DTI) dataset. The V1 image is the vector image for principle tensor direction and colors indicate the direction of vector (red: right–left, green: anterior–posterior, blue: superior–inferior). For each subject, the hand fiber tracts were extracted using two ROIs that were placed on the **(A,C)**. The foot fiber tracts were used **(B,C)**.

For the DTI data, fiber tracking was performed with the FMRIB diffusion toolbox (FSL; www.fmrib.ox.ac.uk/fsl), which was based on a probabilistic tractography method (5000 streamline samples, 0.5 mm step lengths, curvature thresholds = 0.2) (Kwon et al., 2011a,b; Tournier et al., 2011). Moreover, 12 parameters based affine multi-scale two-dimensional registration process was applied to correct the image distortion due to eddy current using fMRIB diffusion toolbox. The two ROIs for extracting the CSTs of each subject were defined in the lower portion of the pons and fMRI activated area (Figure 1). In order to avoid location inaccuracy by individual variation of the brain, extracted individual CSTs for hand and foot were normalized on the Montreal Neurological Institute (MNI) template by using the transformation matrix obtained from the normalization of the *b* = 0 images. The locations of hand and foot fibers, which showed the highest probability values in the CR, were found using ImageJ (Wayne Rasband, NIH, USA) (Mori and van Zijl, 2002; Hong et al., 2010; Kwon et al., 2011b).

**Figure 2:**
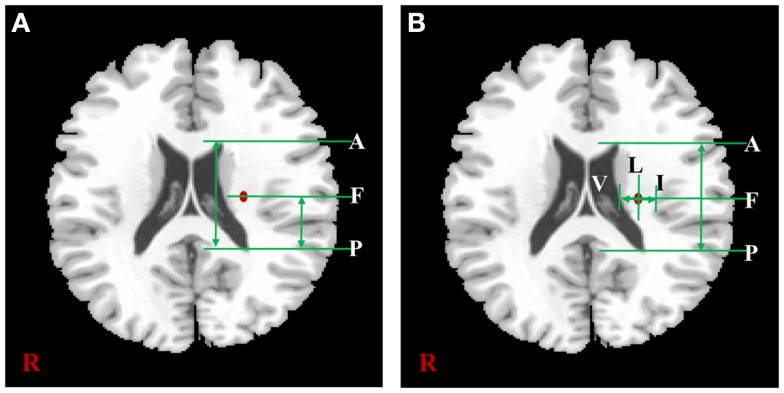
**The distance measurement procedures referred to two previous studies**. According to the study of Kim et al. **(A)** is shown the measurement procedure of anteriority distance percentage ratio based on the lower CR template image, and can be described as $\frac{\mathrm{CP}}{\mathrm{AP}}\times 100$. According to Song’s study, **(B)** is shown the measurement procedures of anteriority and laterality index based on the lower CR template image. In this image, the anteriority index can be described as $\frac{\mathrm{CP}}{\mathrm{AP}}$, and laterality index can be described as $\frac{\mathrm{VI}}{\mathrm{VL}}.$ In both **(A,B)**, “A” is anterior pole, and “P” is posterior pole of the lateral ventricle; “V” is the wall of lateral ventricle, and “I” is the gray matter margin of the cortex; “F” is the location where highest probability value of fiber.

The measured locations of each subject were used for a comparative assessment with previous lesion studies. In the two previous studies, both the hand and foot movements and related lesions in patients with CR were measured in the actual location. The same location measurement methods mentioned in these previous studies were applied in this study. The measurement procedures based on the template image were shown in Figure 2. First, in Figure 2A, we measured the anteriority of hand and foot fibers in the lower and upper CR by reference to Kim and Pope (2005), which measured the distance percentage ratios between the center of the hand and foot fibers and the posterior pole of the lateral ventricle, and between anterior and posterior pole of the lateral ventricle. Second, in Figure 2B, we measured anteriority and laterality index of hand and foot fibers in the lower CR by reference to Song’s study (Song, 2007). The anteriority index was measured by the distance ratio between anterior and posterior pole of the lateral ventricle. Also, the laterality index was measured by the distance ratio between the wall of the lateral ventricle and the margin of gray matter on the insular cortex (Song, 2007).

## Results

**Figure 3:**
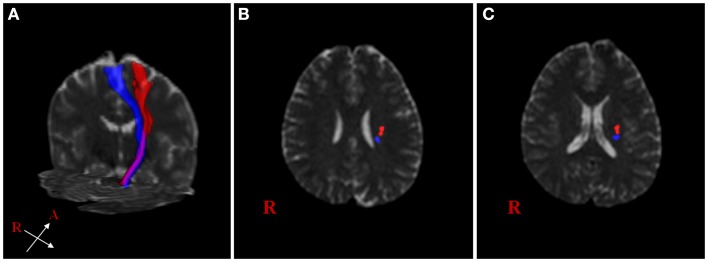
**Reconstructed hand and foot fiber tracts for an individual subject**. **(A)** shows both fiber tracts (red: hand fiber tract, blue: foot fiber tract). **(B,C)** show both fiber tracts overlaid on transverse non-diffusion-weighted images at upper and lower corona radiata (CR) level, respectively.

**Figure 4:**
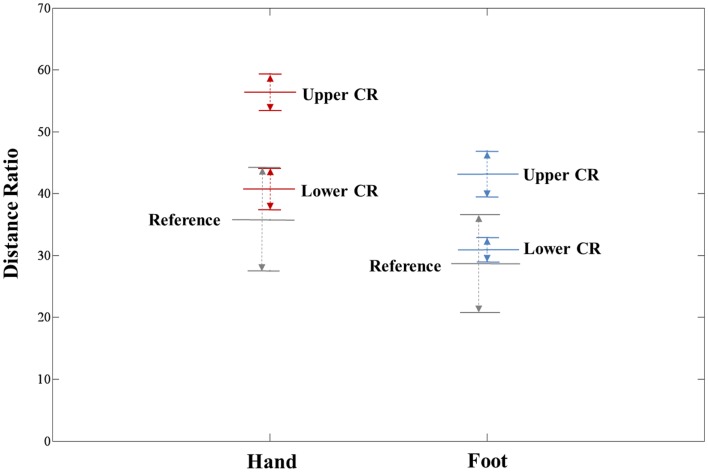
**Anteriority measurement results for hand and foot fibers in the upper CR and lower CR compared with the lesion study of Kim and Pope (2005)**. The distance ratio is represented as mean ± SD. The red line, blue line, and gray line indicate the hand fiber, foot fiber, and reference location values, respectively.

**Figure 5:**
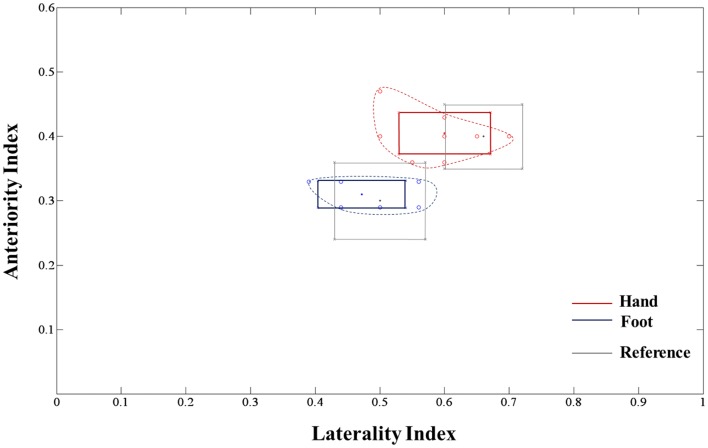
**Anteriority and laterality measurement results for hand and foot fibers in the lower CR compared with the lesion study by Song (2007) are shown**. The red line, blue line, and gray box-shaped line indicate the hand fiber, foot fiber, and reference anteriority/laterality index mean ± SD values, respectively. The dotted line, which links red and blue circle points, represents the margin of individual index values.

The extracted hand and foot fiber tracts are represented in Figure 3. The distance ratios relating the CSTs of the hand and foot to the posterior pole of the lateral ventricle and anterior to posterior pole of the lateral ventricle is shown in Figure 4. The mean ± SD of normalized hand and foot fibers in the upper CR for each subject were represented at 56.40 ± 2.95 and 43.2 ± 3.68, respectively. In the lower CR, hand and foot fibers were represented at 40.72 ± 3.34 and 30.90 ± 1.91, respectively. The results show that hand fibers are located anterior to foot fibers in the upper and lower CR. Especially in the lower CR, our results showed similar values as compared with the lesion study of Kim and Pope (2005). In the reference research, the lesions of hand and foot were represented at 35.80 ± 8.30 and 28.70 ± 7.90, respectively. The results corresponded to our results within the margin of error. According to Song’s study, the measurements of the anteriority and the laterality of the motor fibers are represented in Figure 5. The location measurements were analyzed in lower CR because the results shown in Figure 4 matched better in lower CR than Upper CR. The anteriority index of each subject’s hand and foot fibers in the lower CR were represented at 0.40 ± 0.03 and 0.31 ± 0.02, respectively. The laterality indices of hand and foot fibers were represented at 0.60 ± 0.07 and 0.47 ± 0.06, respectively. These results were concord very well with the study of Song (2007).

## Discussion

Brain white matter consists of axons that connect many functional regions of the brain. There are many studies about these fiber tracts such as confirmation of accurate anatomical location and patient rehabilitation. Especially, many of these studies were performed using DTT technique. The DTT is generally used to investigate brain connectivity based on the premise that the output of tractography algorithms is a reflection of the corresponding white matter tracts and their connectivity status. Most tractography algorithms in common use rely on line propagation techniques based on deterministic tractography algorithm. These algorithms rely on the identification of a suitable position to initiate the seed point and the propagation of the track along the estimated fiber orientation, which is arranged by major eigenvector of the diffusion tensor. Therefore, the deterministic tractography algorithm quickly calculates the direction of the fiber tract, which gives a great advantage. However, there are limitations of this technique for fibers superimposed regions such as crossing fiber regions because deterministic tractography algorithms only provide a single estimate of the path of white matter fibers from each supplied seed point. On the other hand, probabilistic algorithms attempt to avoid this limitation by providing their results in the form of a probability distribution, rather than a single estimate. The fundamental difference is the use of white matter orientation estimates that are drawn from the local probability density function (PDF) of fiber orientations (Mori and van Zijl, 2002; Behrens et al., 2003; Parker et al., 2003; Tournier et al., 2011). The main benefit of probabilistic approaches compared to the deterministic algorithm is that they can provide an estimate of the precision with which a tract pathway has been reconstructed and it leads to increase the probability of fiber progress directions. Both methods have a common limitation as manual guidance of ROI based fiber tracking. Therefore, researchers also used functional MRI results as ROI setting when they performed fiber tracking using DTI data to minimize the user-dependent erroneous factor (Yamada et al., 2007; Han et al., 2010).

In this study, we used above mentioned techniques to evaluate the somatotopic locations of the CST for the hand and foot in the human brain CR. We also used methods of previous lesion studies for location measurements in the CR and our results were comparable with theirs. The CR is an important part of the treatment of stroke patients because many lesions are located in CR. In the case of stroke patients, ability to exercise is often limited and it is directly connected to the damage of neural fiber tract related with movement. Therefore, previous lesion studies were also studied patients with lesions in the CR, and movement locations were evaluated on the basis of the lesion location. The measured results using DTT are more specific and precise than results of previous lesion studies. This is thought to be due to several limitations in the previous lesion studies. The previous lesion studies used a user-dependent location measurement method and they did not consider the individual variation of each patient’s brain. Additionally, they did not consider CR levels in the human brain. Although direct comparisons between our results and previous lesion studies have some difficulties, acquisition of accurate anatomical information using DTT is an important feature of this study.

There are several limitations identified in this study. First, image acquisition using 1.5 T MRI system is relatively limited in technology compared with 3.0 T/7.0 T MRI system. For future studies, we plan to operate a high-field MRI system (≥3.0 T) to give a better comparison to the other studies. Second, limited number of subjects was studied. Although our results showed a good agreement and similar tendency on the location measurement, it would give more reliability to the study if larger number of subjects were participated. Finally, we used highest probability value points to measure the CST location. Although this approach is reasonable to measure the location, using a point with coordinate has a limitation on representing the locations through the image. In a future study, we will use the fiber tractography results of each subject at CR to make a probability map. Then, the probability map will be an image of each subject’s fiber tract overlaid at the same slice, and it will be more helpful to intuitively identify the CST location in the brain.

In conclusion, our results showed that the tractography of hand and foot fibers were in good agreement with previous lesion studies. We also showed that the hand and foot fibers were separated in CR on their accurate locations without erroneous factors. Typically, the patients who had brain injury and disease such as stroke were frequently affected in the motor fibers in the CR (Kim and Pope, 2005; Song, 2007). The exact locations of the motor fibers in the CR are thus very important for patient rehabilitation and follow-up study. From this point of view, our methods and results may be used as a standard for DTT combined with lesion location studies in patients who need rehabilitation or follow-up.

## Conflict of Interest Statement

The authors declare that the research was conducted in the absence of any commercial or financial relationships that could be construed as a potential conflict of interest.
